# The Diagnosis and Therapy of Osteoporosis in Gaucher Disease

**DOI:** 10.1007/s00223-024-01340-y

**Published:** 2025-01-22

**Authors:** Gemma Marcucci, Maria Luisa Brandi

**Affiliations:** 1 Department of Biomedical, Experimental and Clinical Sciences, University of Florence, Bone Metabolic Diseases Unit, University Hospital of Florence, Florence, Italy; 2 Fondazione FIRMO Onlus, Italian Foundation for the Research On Bone Diseases, Florence, Italy; 3 Donatello Bone Clinic, Villa Donatello Hospital, Sesto Fiorentino, Italy

**Keywords:** Gaucher disease, Bone, Osteoporosis, Osteopenia, Bone mineral density, Bone complications

## Abstract

Gaucher disease is a rare lysosomal storage disorder characterized by the accumulation of glucocerebroside lipids within multiple organs due to a deficiency of the lysosomal enzyme (acid β-glucosidase). It is an inherited autosomal recessive disease. The onset of symptoms can vary depending on disease type and severity, with milder forms presenting in adulthood. The main clinical manifestations include cytopenia, splenomegaly, hepatomegaly, and bone lesions. GD is characterized by several bone manifestations, such as osteopenia/osteoporosis, focal lytic or sclerotic lesions, osteonecrosis acute or chronic bone pain, Erlenmeyer flask deformity, and subchondral joint collapse with secondary degenerative arthritis. In 70–100% of patients affected by Gaucher disease type 1, clinical or radiographic evidence of bone disease occurs. Among bone complications, osteoporosis is very common, but its etiopathogenesis in GD is not completely clear. Results deriving from experimental studies support the hypothesis that there is an aberrant activity of both osteoclasts and osteoblasts due to several factors, resulting in impaired bone turnover. Bone complications represent the main cause of pain, disability, and reduced quality of life in these patients. Therefore, there is a need to enhance awareness among physicians on the skeletal manifestations throughout life of GD patients, in order to improve diagnosis and management of bone complications. In particular, this narrative review focuses on risk of bone fragility in GD, etiopathogenetic hypotheses, epidemiological data, diagnosis, monitoring, and treatment of osteoporosis in patients suffering from Gaucher disease, specifying the challenges not yet addressed.

## Introduction

Gaucher disease (GD; ORPHA:355) is a rare lysosomal disorder leading to lipid accumulation (Glucosylceramide and Glucosylsphingosine) in several organs, causing cytopenia, splenomegaly, hepatomegaly, and bone lesions [1]. In GD, bone complications are among the most prevalent clinical manifestations and they represent a major cause of pain, disability, and reduced quality of life in these patients [2].

The diagnosis of GD can be confirmed by evaluating the deficiency of acid glucocerebrosidase activity in leukocytes. GD is inherited in an autosomal recessive manner [3]. It is caused by mutations in the *GBA1* gene, located on chromosome 1 (1q21), leading to a variable decreased activity of the glucocerebrosidase lysosomal enzyme, which hydrolyzes glucosylceramide into ceramide and glucose [1]. In very rare cases, GD can also be caused by a deficiency of the glucocerebrosidase activator, called “saposin C” [4]. Although GD is a rare disease, it is one of the most common lysosomal storage disorders [3].

GD encompasses a continuum of clinical manifestations, from a perinatal-lethal to an asymptomatic adult form. Traditionally, GD is characterized by three major clinical types called 1, 2, and 3, and two other clinical forms (perinatal-lethal and cardiovascular types). Each GD clinical type has different prognoses and management [3]. The three major clinical forms are characterized by the absence in type 1 or presence in types 2 and 3 of primary central nervous system involvement [3]. The most frequent form of GD in Europe and the Americas is the adult non-neuronopathic form (GD type 1) [3]. In GD, there is not a clear correlation between genotype-phenotype; indeed, differences in phenotype have been reported between monozygotic twins. Moreover, the factors that influence disease severity or progression within particular genotypes are not known [3, 5, 6].

Epidemiological data for GD are scarce, except for specific populations. Prevalence estimates indicate a higher prevalence in populations or regions with known founder variants, such as Ashkenazi Jews [3]. The incidence of GD type 1 was estimated at 0.45–22.9:100,000 live births in Europe and North America, type 3 GD at 1.36:100,000 live births in Asia–Pacific, and in Ashkenazi Jewish descendants, prevalence may reach 0.05–0.15% [3].

GD is characterized by several bone manifestations, including osteopenia/osteoporosis, pathologic fractures, focal lytic or sclerotic lesions, osteonecrosis acute or chronic bone pain, bone crises, subchondral joint collapse with secondary degenerative arthritis, and Erlenmeyer flask deformity [2].

Bone complications occur more frequently in the adult form of GD type 1, which is less severe than the others. Indeed, in 70–100% of patients affected by type 1 GD, clinical or radiographic evidence of bone disease is reported, and osteoporosis is a very frequent complication [3]. Bone complications may not correlate with the severity of hematologic or visceral manifestations of GD [3].

Therapeutic options for GD include enzyme replacement therapy (ERT) or substrate reduction therapy (SRT), and hematopoietic stem cell transplantation may be an option for patients affected by severe GD with chronic neurologic involvement [3]. ERT and SRT can also improve bone complications, albeit more slowly over time. To date, few data have been published on the use of anti-fracture drugs in case of osteoporosis in patients with GD.

This review describes the main bone complications in GD, then focusing on osteoporosis, describing its pathogenesis, epidemiology, diagnosis and treatment, and highlighting the main challenges of the disease, which should be addressed in future studies.

## Methodology

A narrative review of available literature was carried out. Papers were retrieved by means of a PubMed enquiry (from 1996 up to June 2024) using the following terms: “Gaucher disease”, and “bone or skeletal complications”, “osteoporosis”, “bone loss”, “bone fragility”, “fractures”, “bone mineral density”, “bone turnover markers”, “enzyme replacement therapy”, “substrate reduction therapy”, “bisphosphonates”, or active principles of bisphosphonates (such as alendronate, risedronate, zoledronate), “denosumab”, “romosozumab”. Randomized controlled studies, observational studies, case-controlled studies, cohort studies, systematic reviews, meta-analyses, narrative review, expert consensus, guidelines, case series and case report were searched. Only English-language papers were reviewed. Letters, comments, editorials, and personal communications were excluded. Main review articles on this topic were retrieved and appropriately cited.

### Bone Complications in Gaucher Disease

In GD, bone manifestations include: osteoporosis/osteopenia, fragility fractures, Erlenmeyer flask deformity, osteonecrosis, lytic bone lesions, bone crises, osteosclerosis, cortical thinning, rarely acute osteomyelitis, and growth retardation [7]. More than half of patients with GD type 1 have a radiological manifestation of bone disease (marrow infiltration, Erlenmeyer flask deformity, or osteonecrosis), as described in the large group of patients (n: 2004) with GD type 1 enrolled in the International Collaborative Gaucher Group (ICGG) Gaucher Registry, from 1991 to 2000 [8]. The Gaucher Registry is a global registry that collects clinical, demographic, genetic, biochemical, and treatment characteristics of GD patients [8]. In patients affected by GD, bone manifestations are prevalent at all ages, and may progress asymptomatically despite effective disease‐modifying treatment for other GD manifestations [2, 8–10].

The pathogenesis of bone complications is not completely clear; however, it is known that the bone marrow and the mineralized components of bone are affected [2]. Different processes are involved, including: bone marrow infiltration and plasma cell dyscrasias; alteration of bone turnover (modeling and remodeling) with loss of bone mineral mass, cortical thinning, lytic lesions, and fragility fractures; osteonecrosis and/or osteosclerosis, cortical infarcts, joint destruction and deformities [2]. Histological studies conducted on bone tissue have shown, in addition to the presence of Gaucher cells (enlarged macrophages containing unprocessed glucocerebroside), osteolysis, necrosis, fibrosis, loss of trabecular bone, and deficit of hematopoiesis remodeling [11]. GD cell infiltrations induce many changes that can affect bone tissue, including impairment of vascularity, increase of intramedullary pressure, alteration of the immune environment, hematopoiesis, and bone cell function [11].

Erlenmeyer flask deformity (EFD) is characterized by a lack of the typical concave di-metaphyseal curve, resulting in a progressive enlargement of the metaphyseal area, due to local bone marrow infiltration by Gaucher cells. EFD usually occurs before puberty, and the sites affected include the distal femur and the proximal tibia or humerus. EFD in the distal femur is often described in adults, although they are asymptomatic and not pathognomonic for GD [7, 12].

Focal osteolytic lesions are uncommon small erosions, characterized by rarefied cortex with dentate endosteum, frosted glass, or favus aspect. Osteolysis is associated with expanding lesions that typically cause cortical thinning and pathological fractures of the shaft of long bones [12]. These lytic lesions are probably caused by the action of cysteine proteases secreted by Gaucher and related cells [13].

Osteonecrosis is likely another consequence of the infiltration of bone marrow by Gaucher cells [14]. Osteonecrosis is not uncommon, in fact, in a UK cohort it is described in up to 43% of adult GD patients [15]. These lesions are often multifocal, and new lesions can occur at any time throughout life and may be associated with puberty or pregnancy [16]. It commonly causes chronic pain and disability and may require surgical intervention [17]. Osteonecrosis is often predicted by a bone crisis [7]. Bone crises are an acute form and are characterized by severe bone or joint pain, often associated with fever, edema, and local and systemic inflammation. Infectious osteomyelitis can be placed in differential diagnosis with bone crises, but in the latter there is no bacteremia [18]. Magnetic resonance imaging, but also radionuclide bone and bone marrow scintigraphy, help early on to distinguish a bone crisis from infectious osteomyelitis (1–3 days after pain onset). Initially in GD bone crises, radionuclide bone scans are usually “cold”, but “hot” in case of osteomyelitis [2].

Finally, a very rare skeletal complication of GD is an extraosseous extension of Gaucher cells that can occur after cortical destruction (i.e. during surgical procedures) with leakage of infiltrates into adjacent tissue [19]. These lesions may be preceded by cortical thinning caused by increased local pressure, altered vascularity, and increased cortical porosity for cytokines activating osteoclasts [19].

GD specific treatments (ERT/SRT) appear to mitigate several of these complications, such as bone marrow infiltration by pathological macrophages and reduced bone mineral density (BMD). On the other hand, secondary complications like osteonecrosis, or tertiary complications like joint complications (osteoarthritis, arthrosis), or bone deformity after fragility fractures, are obviously not reversed by specific GD treatment [11].

### Osteoporosis in Gaucher Disease: Epidemiology

Data analysis from the ICGG Gaucher Registry of patients, treated with imiglucerase between the ages of 5 and 50 years, showed that low decreased BMD was prevalent in all age groups, and the average lumbar BMD Z‐score, measured with Dual-energy X-ray absorptiometry (DXA), was approximately − 1.0, but with a wide distribution in adult patients [20]. In particular, Z-score was < − 1 in 44% of children (*n* = 43), 76% of adolescents (*n* = 41), 54% of young adults (*n* = 56) and 52% of older adults (*n* = 171). Low BMD was most striking in pre-treated adolescents [20]. In GD type 1, the reduction of BMD below the expected range for chronological age (Z‐score < − 2.0) usually occurs early and may become apparent by the age of 5 years [20]. The most common *GBA1* (glucosylceramidase beta 1) genotype was N370S heteroallelic [20]. A previous study conducted on 61 adult patients affected by GD type 1 showed that the reduction of BMD correlated significantly with other clinical indicators of disease severity, such as the N370S/84GG genotype, prior splenectomy, and hepatomegaly; however, the association with splenectomy or whether it was due to greater disease severity was not clear [21].

The ICGG Gaucher Registry described that reduced lumbar BMD appeared to be associated with a high risk of fracture of the spine or femur in patients with GD type 1 [2, 22, 23]. This registry described fracture histories in 319 out of 1552 (20.5%), but without a difference (*p* = 0.8519) in GBA1 genotype distribution between the groups of patients with and without fractures. In GD type 1 patients, the most common site of fracture was the spine (36.4% of all fractures, 119/327), followed by the lower extremities, such as distal to the knee, femur, hip, knee. The described fractures usually occurred at a relatively young age (range age: 34.1–56.9 years) [2, 22]. The 42.3% of fractured patients were untreated at the time of the index date, similar percentage (43.7%) to non-fractured patients. Cases of fractures were identified based on affirmative radiographic or MRI reports in the ICGG Registry. For each patient, the authors reviewed skeletal history and individual skeletal assessments reported to the Registry, and identified all patients who had not suffered avascular necrosis or fractures, and these patients served as controls after appropriate matching. However, it is possible that the fracture prevalence in GD patients collected in the ICGG registry was underestimated, because asymptomatic vertebral fractures were not recorded [2, 22]. Moreover, it is not specified whether vertebral morphometry X-ray was used and it is not described whether the fractures were traumatic or atraumatic.

In an observational, cross-sectional study, conducted on 100 adult patients (range age: 19–85 years, median age: 49 years; female sex: 60%) affected by GD (96/100: GD type 1), fragility fractures were reported in 28% (cumulative incidence), a percentage only slightly higher than in the study by the ICGG Gaucher Registry [15]. At the time of the study, ninety-two patients were receiving ERT (median dose was 30 U/kg per month, and median duration was 8.5 years). Unlike the ICGG Gaucher Registry, in this study a plain radiologic skeletal survey was performed in all patients to document the presence of vertebral and nonvertebral fractures, and fragility fractures were defined as fractures (including vertebral fractures) occurring in the context of little or no trauma [15]. Fragility fracture was associated with younger age at presentation, severity score index, and splenectomy, but not with current age or Erlenmeyer flask deformity [15]. Fragility fracture was associated with forearm T-score, but was not significantly associated with BMD at other sites [15]. Another observational study conducted in a cohort of adult French GD type 1 [n:105, mean (SD) age: 45.6 (13.7) years], in 45 centers distributed widely across France, reported a prevalence of vertebral fractures equal to 15% (seven women and nine men), among them five patients had multiple vertebral fractures. [24]. Most fractured patients (87.5%) were on ERT, one on Miglustat and one without treatment. Fractures described were in almost all vertebrae (from T3 to L5 inclusive). Standard spine X-rays, quantitative CT, or MRI evaluations of the spine were available for central assessment and semiquantitative grading of vertebral fractures in a total of ten patients. Most of these fractures occurred spontaneously or after minimal trauma (even those classified as Genant grade 3), at a mean age of 47 years (range 29–70). Patients ith vertebral fractures had a history of more severe bone involvement, with a greater number of bone complications. However, morphometric vertebral radiological examination was not performed at the level of the dorsal and lumbar spine in all patients. Therefore, the number of vertebral fractures may have been underestimated in this study [2, 24].

Finally, a study from the ICGG Gaucher Registry confirmed a correlation between BMD and risk of fragility fractures, describing that Z‐score ≤ − 1 in GD patients increased the risk of a fragility fracture by a factor of 5 [20, 25, 26]. However, hip BMD was somewhat more strongly related to most of the fracture types than spine or peripheral BMD measures. This correlation resulted significantly higher compared to that commonly described in osteoporotic fracture in population studies [20, 25, 26].

Although data regarding osteoporosis and risk of fragility fracture evaluation in the population affected by GD are still scarce, studies by the ICGG Gaucher Registry and some cohort studies conducted on BMD evaluation and bone fractures in GD patients show that osteoporosis is a common bone complication, although the risk of fragility fractures needs further study. However, in some clinical cases, there could be a relative stability of bone manifestations, in addition to hematological indices, organ volumes, and markers of disease severity, even without specific treatments, as recently described in a small retrospective, observational study conducted in 31 adult patients with GD1 of both Ashkenazi-Jewish (AJ) and non-AJ ancestry for a median follow-up duration of 15 years [27].

### Osteoporosis in Gaucher Disease: Pathophysiology

Osteoporosis or osteopenia are very common bone complications in GD, especially in GD type 1, as already mentioned. Etiopathogenesis of reduced BMD in GD is unclear, however, results deriving from experimental studies support the hypothesis that there is both osteoclast and osteoblast aberrant activity, caused by several factors, resulting in impaired bone turnover (imbalance between the bone formation and resorption process) [28].

In GD, mesenchymal stem cells may be adversely affected by lipid accumulation, compromising osteoblastogenesis [29]. Studies with ex vivo cell cultures and in vivo experiments in mice described that glucocerebrosidase‐deficient mesenchymal stromal cells (nonhematopoietic osteoblast precursors in the bone marrow isolated from 10 patients with GD type 1) had impaired ability to proliferate and differentiate into osteoblasts [2, 30–32]. On the other hand, studies on tissue culture showed a more rapid differentiation into osteoclasts arise from glucocerebrosidase‐deficient peripheral blood mononuclear cells (PBMCs), compared to conditions with monocytes with normal glucocerebrosidase activity [2, 30]. In vitro studies have also shown that the bone resorptive activity of glucocerebrosidase‐deficient osteoclasts is enhanced compared to normal osteoclasts [2, 30].

A study conducted on adult patients with GD (n:39) and a group of healthy controls showed that the majority of patients with elevated sclerostin levels (an inhibitor of osteoblastogenesis) also had osteopenia or osteoporosis, and the balance between sclerostin and DKK-1 had waned, suggesting that the Wnt signaling pathway might play a role in GD-associated bone disease [33].

Finally, a recent study on an in vitro model of GD patient cells described that elevated endoplasmic reticulum stress and inflammasome activation are indicative of osteoporosis development [34].

Until now, the evaluation of the role of bone turnover markers in osteoporosis and other bone complications has only partially been investigated in GD, and the findings described in literature are inconclusive [7]. This is probably due to differing experimental conditions and factors interfering with the measurement of bone turnover markers, such as: age, pubertal development, and other interfering treatments. Regarding the typical markers of bone formation and resorption, a case‐control study conducted on children and adolescents with untreated GD showed that serum osteocalcin levels (a marker of bone formation) and type 1 collagen C‐terminal telopeptide (a marker of bone resorption) were lower compared to controls, suggesting a low bone turnover [35]. During skeletal growth, the impairment of bone remodeling in GD type 1 may cause an inadequate peak bone mass, but it is not known whether there is a mechanistic link between pubertal delay in GD and the suppression of bone turnover [2, 32]. On the other hand, a study conducted on adult patients affected by GD type 1 (n:12) showed that levels of cathepsin K (a protein secreted by osteoclast) were higher compared to healthy control subjects (n:26) [13, 36]. Since cathepsin K is highly active in bone resorption, it could be a marker indicative of bone resorption in adult GD patients, although it is not currently used in clinical practice [37].

Regarding the potential use of a specific GD marker, serum glucosylsphingosine levels may be an indirect pathologically relevant biomarker for osteoporosis, as it appears to mediate osteoblast dysfunction in GD [2, 29].

A widespread deficiency of 25 oh vitamin D in GD patients has been described, although it is also reported in the general population. In particular, Mikosch et al. reported low 25‐OH vitamin D levels in 83% GD patients in the UK (total number patients: 60) at several points throughout the year [2, 38].

In conclusion, further studies are necessary to confirm the results described and to complete our knowledge of the etiopathogenesis of the onset of osteoporosis in patients affected by GD.

### Bone Quantity and Quality Assessment in Gaucher Disease

In 2019, an expert panel consensus pointed out that, in GD patients, evaluation of BMD by DXA may be complicated by focal anatomical changes and focal bone alterations associated with this disease [2]. The DXA assessment at the level of the femoral head may not be possible in case of osteonecrosis with subsequent sclerotic and arthritic processes. Given that osteonecrosis can also occur within the vertebral bodies in GD, causing an area of sclerosis, this may overestimate the BMD measurement at this site. Alterations of water and fat composition of the marrow may also affect the measured BMD values, and the assumptions that are usually made regarding the marrow fat content may not be valid in GD patients. Moreover, in case of vertebral fractures, the affected vertebrae should be excluded in BMD assessment, as in the general population. Other factors that could interfere with the measurement of BMD by DXA in terms of overestimation include osteophytes and extraskeletal calcifications (vascular calcifications, and calculi) [2].

Quantitative computed tomography (QCT) of the lumbar spine, a radiological technique that measures BMD using a standard X-ray computed tomography, is not usually recommended in order to avoid unnecessary radiation exposure, except for cases where it is necessary to assess critical cortical‐thinning associated with lytic lesions or resolve not easily interpretable DXA results [2].

The DXA exam usually measures only bone mineral quantity, however, both bone mineral quantity and bone quality, resulting from bone microarchitecture, geometry, and turnover, contribute to bone strength and risk of fragility fractures [39]. In general, bone quantity accounts for approximately 60–70% of bone strength and bone quality for 30–40%. In particular, altered bone microarchitecture is an important factor in accounting for fragility fractures, independently of BMD [39]. Therefore, in GD, as in other forms of secondary osteoporosis, the assessment of bone quality is likely to be fundamental.

Regarding bone microarchitecture assessment, trabecular bone score (TBS) is a novel tool that provides information about trabecular microarchitecture, because it is correlated with microarchitecture and spatial arrangement of the trabeculae, therefore it is a validated “index” of bone microarchitecture [39–41]. It is an imaging technology adapted directly from DXA images of the lumbar spine. Recently, several studies have shown that low values of TBS predict significantly current and future fragility fractures in primary osteoporosis, and some studies have also demonstrated its potential utility in secondary osteoporosis, irrespective of densitometric findings [39, 42, 43].

In 2021, an observational cross-sectional study evaluated bone involvement in 32 adult patients with GD type 1 (mean age: 40 ± 16 years) undertreated with ERT using TBS in addition to standard imaging, such as X-rays, BMD measurements by DXA, and magnetic resonance imaging [44]. Patients had received velaglucerase for 2.7 ± 1.4 years and more than half of the patients (*n*:19/32) had been treated previously with imiglucerase. Only 18.7% of patients had abnormal BMD, with no correlation with fractures. TBS values resulted to be less than 1350 score (indicative of partially degraded/degraded microarchitecture) in 53% of patients, showing greater degradation of bone quality than of bone mass, and tended to be lower in those with fractures (*p* = 0.06). Therefore, bone quality evaluated by TBS was more affected than BMD in GD patients with fractures [40, 44]. Moreover, since Khan et al. showed that GD patients with Z-scores of lumbar spine (DXA) < − 1 had a higher risk of fractures [22], patients of this study with Z-score < − 1 were analyzed separately, and the authors described this group as having significantly lower TBS values compared to patients with Z-score ≥ − 1 [44].

A previous study evaluated both altered trabecular microarchitecture by TBS evaluation and Hip Structural Analysis (HSA) in 11 GD type 1 patients (median age 22 years, range 3–73) [45]. Low levels of BMD were described in half of the patients, but this parameter was not associated with pathological fractures. TBS, instead, resulted significantly lower in patients with pathological fractures (*p* = 0.024); therefore, this score appears to be a potentially useful tool, improving the assessment of GD-related the risk of fragility fractures. Moreover, lower TBS levels were described in patients with avascular osteonecrosis (*p* = 0.049), but could not identify those with bone infarctions. The relationship described between TBS and osteonecrosis could be explained, considering that the necrotic/infarcted bones have an irregular distribution of the bone spiculae, leading to a low TBS value. Among HSA parameters, the Cross Sectional Area of the intertrochanteric region and the Buckling Ratio of the narrow neck, another feature associated with mechanical resistance to stress, allowed the distinction of patients with avascular osteonecrosis, although HAS could be influenced by bone degeneration due to previous femoral ischemic events [46].

A study cohort study evaluated the utility of DXA and radiographic femoral cortical thickness measurements as predictors of fragility fracture in patients affected by GD with long-term follow-up (the median follow-up period was 11 years; IQR, 7–19 years; range, 2–30 years) [47]. In the 212 GD patients without baseline fracture, Cortical thickness index (CTI) (cutoff, ≤ 0.50) predicted future fractures with higher sensitivity and specificity (area under the receiver operating characteristic curve [AUC], 0.96; 95% CI: 0.84, 0.99; sensitivity, 92%; specificity, 96%) than DXA T-score at total hip (AUC, 0.78; 95% CI: 0.51, 0.91; sensitivity, 64%; specificity, 93%), femoral neck (AUC, 0.73; 95% CI: 0.50, 0.86; sensitivity, 64%; specificity, 73%), lumbar spine (AUC, 0.69; 95% CI: 0.49, 0.82; sensitivity, 57%; specificity, 63%), and forearm (AUC, 0.78; 95% CI: 0.59, 0.89; sensitivity, 70%; specificity, 70%). The authors concluded that CTI ≤ 0.50 appeared to have an optimal accuracy in predicting the bone health status and the risk of fracture in GD as independent predictor [47]. Therefore, further studies are needed on the use of CTI to monitor disease progression and/or response to treatment in GD patients.

A cross-sectional study including 16 patients with GD type 1 selected from the Catalan Study Group and 29 age- and sex-matched controls also described altered bone strength measured by impact microindentation [48]. Bone Material Strength index (BMSi) was measured by bone impact microindentation using an Osteoprobe instrument in the anterior face of the mid-tibia, excluding patients with bone infarcts or any other focal lesion in the area of indentation visible on imaging. Patients with GD type 1 had significantly lower BMSi compared to control group (adjusted beta –9.30; 95% CI, –15.18 to–3.42; *p* = 0.004), and these levels were associated with reduced lumbar BMD (adjusted beta –0.14; 95% CI, –0.22 to –0.06; *p* = 0.002) and total hip BMD (adjusted beta–0.09; 95% CI, –0.15 to –0.03; *p* = 0.006) than the control group. Moreover, BMSi was correlated with chitotriosidase (*p* = 0.004), a serum biomarker of GD activity, because it is an enzyme produced and secreted in large amounts by activated macrophages, especially macrophages loaded with phagocytozed glycosphingolipid in GD. The microindentation could be a tool for assessing and managing their bone health along with other imaging assessments to evaluate bone quality in these patients, although further studies are needed [48].

Therefore, given the limitations relating to the use of DXA in monitoring bone disease in GD, further studies are necessary to validate these new techniques with bone quality assessment, which could improve the prediction of fracture risk in these patients. In addition to those described, another new technique, the high-resolution peripheral quantitative computed tomography (HR-pQCT), which provides a volumetric measurement of BMD and three-dimensional assessment of bone microarchitecture at the distal radius and distal tibia, could be further investigated in GD. To date, its use has only been described in one case report of a patient with GD type 1 who switched from Imiglucerase to Eliglustat [49].

### Fragility Fracture Risk Evaluation in Gaucher Disease

Epidemiological data on fragility fractures are scarce in GD, however some investigations have partly improved our understanding of risk factors for fragility fractures in this population [50].

Overall, fracture risk depends on many factors, as in the general population, including: BMD, bone quality, smoking status, alcohol consumption, physical activities, age, vitamin D deficiency, use of concurrent medications, and comorbidities (e.g., multiple myeloma, steroid use) [2]. However, since the ICGG Registry has already shown that Z-score < − 1.0 in GD type 1 patients was significantly associated with a high risk for fractures (OR, 5.55) at any site [51], unlike the general population, other additional GD specific risk factors for fractures have been hypothesized [22]. In this regard, in 2021 a study investigated whether there were determinants for fracture, other than DXA, easily identified in the GD population after beginning ERT, because fracture risk may be decreased by ERT but not eliminated [51]. Risk fracture scores (e.g., FRAX®) are available for the general population [52], but a tool specific for GD-related factors did not exist [51]. This study evaluated GD type 1 patients enrolled in the ICGG Registry, following patients from GD treatment beginning (or age 18 years for patients if treatment started earlier) to the date of first adult fracture, death, or end of follow-up [51]. The total person-years of follow-up for this analysis population was 30,666 person-years, with individual patients being followed for a median of 10.9 years. During this time, 288 first fractures in adulthood were described (with an overall incidence rate of 9.4 fractures per 1000 person-years). Fifty of the fractures occurred after switching from imiglucerase to another therapy and 84 fractures occurred on or after the beginning of a temporary imiglucerase shortage. The mean (SD) age of first fracture during adulthood for those 288 patients with fracture was 49.2 (16.6) years (range from 18.1 to 90.4 years) [51]. The authors created a composite fracture risk score assessment, called the “Gaucher Risk Assessment for Fracture” (GRAF) for GD patients. This is a clinical tool that analyzes 5 widely available characteristics including: sex, age at treatment initiation (ATI), time interval between diagnosis and treatment initiation, splenectomy status, history of pre-treatment bone crisis, which provides assessment of future fracture risk, when imiglucerase ERT is initiated, regardless of whether there is an eventual switch to an alternative ERT or to substrate reduction therapy. For each ATI group, the GRAF score was associated with a 2.7-fold increased risk of adult fracture for each one-point increase (*p* < 0.02 for < 18 years ATI, *p* < 0.0001 for ≥ 18 years to < 50 years ATI and ≥ 50 years ATI) [51]. Moreover, in case of delays in starting treatment, GRAF score could help predict how much the risk of fracture increases. Indeed, delayed initiation of GD treatment after diagnosis was consistently associated with increased risk for fracture in all adult patients older than 18 years. The percentage of splenectomized patients, in each of the 3 ATI cohorts, resulted higher in patients with fractures than in those without fractures, however, within the group of fractured patients, splenectomy was statistically significantly associated with fracture risk only in the ≥ 18 to < 50 years ATI cohort. Being female resulted to be a significant factor in the ≥ 50 years ATI cohort with presumably the highest proportion of post-menopausal women. These findings suggest that general risk factors for post-menopausal osteoporosis (e.g. smoking, family history) also contribute to the overall fracture risk in female patients with GD type 1. Women of Ashkenazi Jewish ancestry have also been identified as being at high risk for fracture [53]. In this study, 50 of all 288 first-time fractures (17%) occurred after a switch from imiglucerase to an alternative treatment, but the impact of switching treatment on fracture risk is not known [51].

The main limitations of this study were that it is not known whether all fractures were reported in this registry and it was not specified whether they were due to trauma or without trauma. Moreover, ERT dose was not reported. Therefore, in the future this study should be validated on a GD population in which there is an effective assessment of fragility fracture events. Furthermore, it is important to remember that this study analyzed only patients treated with alglucerase and/or imiglucerase, and therefore the GRAF score may not be valid for patients treated with other ERTs [51]. Moreover, GRAF score has not been validated in other populations.

### Osteoporosis in Gaucher Disease: Monitoring and Treatment

In 2018, the European Working Group on Gaucher Disease published an expert consensus document on management goals for GD type 1 including bone manifestations [6, 45]. Regarding bone health, the short-term management goals include an increase of BMD by 2 years in adult patients with a T-score < − 2.5 at baseline and to attain normal or ideal peak skeletal mass in children. The long-term management goals include prevention of bone complications such as pathological fractures, and prevention of osteopenia and osteoporosis (i.e., maintain BMD T-scores > − 1) [6].

In 2019, an expert international group of physicians published useful guidelines for the assessment and management of osteoporosis in GD for practicing clinicians [2]. The authors recommended assessment with DXA of the lumbar spine and left and right hips, considering potentially confounding focal disease, in all adult patients affected by GD [2]. Serial DXA evaluations should consider the Z‐score (or T‐score), BMD, and BMC (bone mineral content) [2]. For children, the posterior-anterior spine and total body less head are the preferred skeletal sites for performing BMC and areal BMD measurements in most pediatric subjects. Other sites may be useful depending on the clinical need [2, 54]. This guideline recommended not using QCT of the lumbar spine to avoid unnecessary radiation exposure; however, it may be helpful in case of unreliable or difficult-to-interpret DXA results due to artifacts and interferences from arthrosis, calcifications or osteonecrosis, and to assess critical cortical‐thinning associated with lytic lesions [2]. Moreover, ultrasonography for the assessment of BMD and bone quality was not recommended [2]. For the assessment of fracture risk, the authors remembered to exclude the influence of comorbidities increasing the fracture risk, such as multiple myeloma, steroid use, etc. [2]. To date, there are no indications relating to imaging exams on the quality of bone tissue in the guidelines, due to the lack of data on their validation in GD.

For bone health in the GD population, adequate daily calcium intake and 25 OH vitamin D levels in the normal range (30–50 ng/ml) should be guaranteed, and GD patients should be treated with calcium and vitamin D supplementation if necessary, according to local guidelines, as for the general population at risk of osteoporosis [2].

Observational investigations and clinical trials conducted on GD patients have described that reduced BMD levels compared by age and sex improve with ERT (imiglucerase, velaglucerase alfa, and taliglucerase alfa) or with SRT (miglustat and eliglustat) [14, 55, 56].

In particular, in 1996, a retrospective analysis conducted on 54 children and adolescents with GD type 1 treated with ERT showed that treatment was effective in normalizing linear growth [57]. Bembi B et al. reviewed other small studies on effects of ERT on growth and BMD in pediatric GD population [58]. Among these, a study conducted on small group of Italian GD pediatric patients treated with ERT (starting with alglucerase and then switching to imiglucerase; the dose ranged from 20 to 45.5 U/kg, given every 2 weeks, with a mean dose of 26.75 U/kg) showed a significant increase in the mean lumbar BMD Z-score after 2 years of treatment (from − 2.17 ± 1.25 at baseline to − 1.57 ± 0.97; *p*:0.003) [58]. Patients with growth delays achieved normal growth rates within 1–3 years after starting treatment. Similar results were obtained in other studies conducted in small groups of pediatric GD patients in Germany and USA [58]. These studies suggested that ERT improves BMD and growth rates in pediatric GD patients [58].

Subsequently, in 2011, Mistry PK et al. examined data on the effects of imiglucerase treatment on BMD, from the ICGG Gaucher Registry of GD patients, treated with imiglucerase between the ages of 5 and 50 years [20]. This analysis showed that imiglucerase therapy for 6 years, in children with DXA Z-scores ≤ − 1 at baseline, improved the mean DXA Z-scores from − 1.38 (95% CI − 1.73 to − 1.03) to − 0.73 (95% CI − 1.25 to − 0.21) and in young adults DXA Z-scores improved from − 1.95 (95% CI − 2.26 to − 1.64) to − 0.67 (95% CI − 1.09 to − 0.26). In older adult GD patients, BMD also improved, but the magnitude of the improvement was lower compared to younger GD patients [20].

In 2014, a phase 2 trial of oral eliglustat (50 or 100 mg), self-administered by mouth twice daily, conducted on 19 adult patients (18–55 years) for 4 years of treatment, showed lumbar spine BMD increased significantly (*p* = 0.02; *n* = 15) by a mean of 9.9% (SD 14.2%) from baseline to fourth year [14]. Mean femur T-score remained normal through 4 years. No lumbar spine or femoral fractures were reported during this study [14].

In 2015, a study reported the results of the 7-year experience of the phase I/II and extension studies conducted on adult GD type 1 patients who received treatment with velaglucerase alfa. Twelve patients were enrolled and received velaglucerase alfa (at a dose of 60 U/kg body weight i.v. every other week) and ten patients completed the phase I/II trial and were eligible to participate in a phase I/II extension study. The effects on BMD were among the exploratory endpoints of these studies. Significant improvements in BMD Z-scores were seen at the lumbar spine by month 24 and at the femoral neck by month 33 and were continuous thereafter [55].

The efficacy of these treatments depends on several factors: the age at diagnosis, the age at start of therapy, the bone affection at beginning of therapy, and the type of bone affection (reversible vs irreversible) [2]. It is essential for ERT or SRT therapy to be started as soon as possible in order to have significant therapeutic effects on bone tissue. Moreover, ERT has been demonstrated to improve BMD, but this requires a very long treatment span [59]. ERT appears to be useful for improvement of BMD in all age groups, although the greatest effect is reported in younger patients before reaching peak bone mass [2, 20]. Several investigations have reported apparent bone mass accretion in children and adolescents after starting enzyme therapy [2, 20, 58]. In the pediatric population and adolescents, the use of enzyme therapy often improves the delayed pubertal development and delayed growth, and therefore it is not clear whether changes in hormonal status and bone growth directly increase BMD; if so, the effect of ERT/SRT would be indirect [55]. However, these studies use differing methods (i.e. technical issues), and therefore their clinical findings are difficult to compare with one another. Moreover, some studies are limited by their retrospective nature.

Unfortunately, until now, no scientific evidence has been published on the anti-fracture effects of therapies specifically used for GD, such as ERT and SRT.

There are very few studies that have evaluated the efficacy of the use of anti-fracture drugs, usually used for osteoporosis, in GD patients treated with enzyme therapy.

A single double-blind, 2-arm, placebo-controlled trial regarding the use of oral bisphosphonate (alendronate, 40 mg/die) was conducted on adult patients with GD (*n*: 24; age range: 18–50 years), treated with enzyme therapy for at least 24 months [60]. This study demonstrated the efficacy of oral bisphosphonate treatment in improving BMD after 18 months, albeit with a dosage not usually used for osteoporosis [56]. However, this dosage was chosen because in previous studies the use of 40 mg of alendronate resulted effective for reducing focal lesions in another bone metabolic disease like Paget disease [61, 62]. Despite the scarcity of published data on the use and efficacy of bisphosphonates in patients with GD, their possible use should be considered, as it is in the general population for osteoporosis. New studies on this topic are desirable and clearly necessary.

So far, the effects of anti-resorptive drugs, such as Denosumab (anti-RANKL monoclonal antibody), have not been established in the GD population [2].

A case report showed that teriparatide, an anabolic drug, 20 mcg/die for 24 months, increased BMD in a 65-year-old woman with GD type 1 (diagnosed at 30 years of age) and a concurrent history of osteopenia and multiple fractures [63]. However, this clinical case was very unusual, because the patient had multiple pathologies, including Sjogren's syndrome and rheumatoid arthritis, and she was also treated with glucocorticoids and did not respond to long-term use of bisphosphonates [63].

Another proposal for osteoporosis in GD could be the use of romosozumab, a monoclonal antibody that binds sclerostin and has a dual effect of increasing bone formation as well as decreasing bone resorption [64]. This drug is a very recent treatment for post-menopausal fracture osteoporosis. There is no data yet on patients with GD.

Finally, some in vitro studies have shown preliminary results on the effects of new therapeutic drugs directed to specific molecular target mechanisms, such as activation of the Wnt/βcatenin pathway by CHIR99021, able to induce bone formation [65]. Moreover, it has been described in in vitro models that two anti-inflammatory drugs, called anakinra and pentosan polysulfate, reduce osteoclastogenesis [66, 67].

Recently, a study evaluated the impact of an integrated therapy comprising ERT, vitamin D (cholecalciferol), and a normocalcemic-normocaloric-hyposodic diet (bone diet) on bone health in 25 adult GD type 1 patients [68]. The authors demonstrated that deficit of 25 OH vitamin D was very frequent in GD type 1 patients and that the cholecalciferol treatment was effective to correct vitamin D deficiency and to reduce PTH levels. Moreover, they showed that the integrated treatment with ERT, cholecalciferol, bone diet, and alendronate 70 mg/week (used only in GD type 1 patients with BMD Z-score ≤ − 2.0) guaranteed BMD stability and the occurrence of no new skeletal manifestations during 2 years of follow-up [68].

Therefore, future studies are necessary to better understand the adequate monitoring of bone fragility in GD, and the effects of pharmacological treatments on the improvement of bone mass and the real anti-fracture effects, in order to make uniform recommendations for these patients.

## Conclusions

Based on studies published to date, it's clear that bone complications, such as osteoporosis/osteopenia, are common in GD, especially GD type 1, and together with the other GD-associated bone complications represent an important cause of pain, disability, and reduced quality of life in these patients [2, 7, 22]. However, there is a need to enhance awareness among physicians on the bone manifestations throughout the life of GD patients, to improve diagnosis and management of bone pathologies [2].

Regarding osteoporosis in GD, future studies should be conducted to further clarify several issues, including: pathophysiology of osteoporosis, fragility fractures epidemiology, evaluation of specific risk factors for fragility fractures, bone quantity and quality evaluation and their predictive role for fragility fractures, effects of ERT/SRT and/or combined with anti-fracture drugs on the reduction of risk of osteoporosis and fragility fractures. The results deriving from future studies conducted on these topics will allow improved prevention and treatment for osteoporosis and fragility fractures also in GD [2, 69, 70]. However, based on the data available to date from studies on GD and secondary osteoporosis, it is advisable, in order to improve the clinical and therapeutic management of osteoporosis in GD patients: (1) in adulthood, to monitor lumbar and dual hip or distal third of the radius (in case of lumbar vertebral collapse or hip osteonecrosis) BMD DXA, every 2–4 years for patients aged < 50 years, including premenopausal women (Z-score); every 12–24 months for patients aged > 50 years (including post-menopausal women) (T-score), extending the frequency if the BMD values ​​allow it; and to evaluate, if available, also bone quality parameters; (2) in childhood (children older than 5 years), to monitor BMD DXA (Z-score) lumbar or total body less head, considering radiation exposure; (3) to monitor mineral and bone metabolism biochemical exams to optimize, in particular, calcium balance and 25 OH vitamin D levels, to evaluate the trend of bone turnover markers, and to evaluate other osteoporosis risk factors; (4) to monitor fracture events with skeletal radiological investigations; (5) to evaluate, if necessary, in adulthood the association of anti-fracture drugs to ERT or SRT therapies, usually used in the most common forms of primary and secondary osteoporosis, although there are very few data described in the literature in GD population [2, 41].
